# Amyloid-β, Tau, and Cognition in Cognitively Normal Older Individuals: Examining the Necessity to Adjust for Biomarker Status in Normative Data

**DOI:** 10.3389/fnagi.2018.00193

**Published:** 2018-06-25

**Authors:** Isabelle Bos, Stephanie J. B. Vos, Willemijn J. Jansen, Rik Vandenberghe, Silvy Gabel, Ainara Estanga, Mirian Ecay-Torres, Jori Tomassen, Anouk den Braber, Alberto Lleó, Isabel Sala, Anders Wallin, Petronella Kettunen, José L. Molinuevo, Lorena Rami, Gaël Chetelat, Vincent de la Sayette, Magda Tsolaki, Yvonne Freund-Levi, Peter Johannsen, The Alzheimer's Disease Neuroimaging Initiative, Gerald P. Novak, Inez Ramakers, Frans R. Verhey, Pieter Jelle Visser

**Affiliations:** ^1^Department of Psychiatry and Neuropsychology, Alzheimer Center Limburg, School for Mental Health and Neuroscience Maastricht University, Maastricht, Netherlands; ^2^University Hospital Leuven, Belgium; ^3^Laboratory for Cognitive Neurology, Department of Neurosciences KU Leuven, Leuven, Belgium; ^4^Alzheimer Research Centre KU Leuven, Leuven, Belgium; ^5^Center for Research and Advanced Therapies CITA-Alzheimer Foundation, San Sebastián, Spain; ^6^Alzheimer Center and Department of Neurology, Neuroscience Campus Amsterdam, VU University Medical Center VU University Amsterdam, Amsterdam, Netherlands; ^7^Department of Biological Psychology VU University Amsterdam, Amsterdam, Netherlands; ^8^Department of Neurology Hospital de la Santa Creu i Sant Pau, Barcelona, Spain; ^9^Section for Psychiatry and Neurochemistry, Institute of Neuroscience and Physiology, University of Gothenburg Sahlgrenska Academy, Gothenburg, Sweden; ^10^Nuffield Department of Clinical Neurosciences University of Oxford, Oxford, United Kingdom; ^11^Alzheimer's Disease & Other Cognitive Disorders Unit, Hopsital Clínic Consorci Institut D'Investigacions Biomediques August Pi I Sunyer (IDIBAPS), Barcelona, Spain; ^12^Barcelona Beta Brain Research Center Unversitat Pompeu Fabra, Barcelona, Spain; ^13^Institut National de la Santé et de la Recherche Médicale UMR-S U1237, Université de Caen-Normandie GIP Cyceron, Caen, France; ^14^Institut National de la Santé et de la Recherche Médicale U1077, Université de Caen Normandie Ecole Pratique des Hautes Etudes, Caen, France; ^15^CHU de Caen Service de Neurologie, Caen, France; ^16^1st Department of Neurology University General Hospital of Thessaloniki AHEPA, Thessaloniki, Greece; ^17^Division of Clinical Geriatrics, Department of Neurobiology, Caring Sciences and Society (NVS) Karolinska Institutet, Stockholm, Sweden; ^18^Department of Geriatric Medicine, Karolinska University Hospital Huddinge Karolinska Institutet, Stockholm, Sweden; ^19^Department of Psychiatry Norrtälje Hospital Tiohundra, Norrtälje, Sweden; ^20^Danish Dementia Research Centre, Rigshospitalet, Copenhagen University Hospital University of Copenhagen, Copenhagen, Denmark; ^21^Janssen Pharmaceutical Research and Development Titusville, NJ, United States

**Keywords:** Alzheimer's disease, amyloid-beta, tau, cognition, neuropsychological examination, normative data

## Abstract

We investigated whether amyloid-β (Aβ) and tau affected cognition in cognitively normal (CN) individuals, and whether norms for neuropsychological tests based on biomarker-negative individuals would improve early detection of dementia. We included 907 CN individuals from 8 European cohorts and from the Alzheimer's disease Neuroimaging Initiative. All individuals were aged above 40, had Aβ status and neuropsychological data available. Linear mixed models were used to assess the associations of Aβ and tau with five neuropsychological tests assessing memory (immediate and delayed recall of Auditory Verbal Learning Test, AVLT), verbal fluency (Verbal Fluency Test, VFT), attention and executive functioning (Trail Making Test, TMT, part A and B). All test except the VFT were associated with Aβ status and this influence was augmented by age. We found no influence of tau on any of the cognitive tests. For the AVLT Immediate and Delayed recall and the TMT part A and B, we calculated norms in individuals without Aβ pathology (Aβ- norms), which we validated in an independent memory-clinic cohort by comparing their predictive accuracy to published norms. For memory tests, the Aβ- norms rightfully identified an additional group of individuals at risk of dementia. For non-memory test we found no difference. We confirmed the relationship between Aβ and cognition in cognitively normal individuals. The Aβ- norms for memory tests in combination with published norms improve prognostic accuracy of dementia.

## Introduction

Neuropsychological examination is an essential element when diagnosing Alzheimer's disease (AD). Normative data for neuropsychological tests enable interpretation of test performance and are typically based on cognitively normal individuals categorized by age, gender and years of education. However, pathological changes related neurodegenerative diseases such as AD, could already be present in aged cognitively normal individuals (Dubois et al., 2016), and could lead to subtle changes in cognition (Vos et al., 2013; Mormino et al., 2014; Jansen et al., 2017). Still, norms for neuropsychological tests do not take AD biomarker status into account, which may lead to underdiagnosis of early stage AD. The aim of the present study was to test whether norms based on individuals without AD pathology, would improve the sensitivity of cognitive neuropsychological tests to identify early AD.

The predominant hypothesis is that beta-amyloid (Aβ) is the first biomarker to become abnormal in AD, followed by neuronal injury markers such as tau (Bateman et al., 2012; Jack et al., 2013). To our knowledge, only one study has investigated the effect of these AD biomarkers on cognitive norms (Hassenstab et al., 2016). In that study, it was found that individuals with normal levels of both Aβ and tau in cerebrospinal fluid (CSF) performed better on cognitive tests, but excluding these individuals with preclinical AD from normative datasets did not increase predictive accuracy of clinical decline in cognitively normal individuals (Hassenstab et al., 2016). However, Aβ and tau may independently impact cognitive performance in the preclinical stage in a test-specific manner. Also, it remains unknown whether biomarker negative norms would increase predictive accuracy of dementia in a memory clinic setting.

Hence in the present study, we first examined the independent effects of Aβ and tau on five frequently used neuropsychological tests in a large sample of cognitively normal individuals. Aβ was measured in CSF or on positron emission tomography (PET) and tau was measured in CSF. Subsequently, we calculated novel norms based on biomarker negative individuals for tests that were associated with AD biomarkers in the initial analyses. Lastly, we examined whether the use of these new norms could improve the identification of individuals at risk of progression to dementia among non-demented individuals from a memory clinic cohort.

## Methods

### Test dataset

#### Subjects

For the test dataset, 907 cognitively normal individuals were selected from eight cohorts of the European Medical Information Framework for Alzheimer's disease (EMIF-AD) project: Barcelona St. Pau (Alcolea et al., 2014; Sala et al., 2017), EDAR (Reijs et al., 2017), Gipuzkoa Alzheimer Project (GAP) (Estanga et al., 2017), Gothenburg MCI study (Wallin et al., 2016), IDIBAPS (Fortea et al., 2010), IMAP+ (La Joie et al., 2014), Leuven (Adamczuk et al., 2015, 2016a,b), EMIF preclinical-AD study (Demuru et al., 2017), and from the American Alzheimer's Disease Neuroimaging Initiative (ADNI) study (Mueller et al., 2005) (Supplemental Table 1). Supplemental Table 2 shows an overview of the participating centers and the included number of subjects.

#### Inclusion criteria

Inclusion criteria were: (1) age above 40 years; (2) no cognitive impairment at baseline (Supplemental Table 2 provides an overview of definitions of CN by cohort); (3) availability of an Aβ measurement in CSF or on amyloid positron emission tomography (PET); and (4) a baseline neuropsychological examination. From the EMIF preclinical-AD study we randomly selected one individual per monozygotic twin pair, to avoid duplication bias.

#### Neuropsychological examination

Neuropsychological examination was performed according to the routine protocol at each site, including the Mini-Mental State Examination (MMSE; Folstein et al., 1975). For the current study we selected the Auditory Verbal Learning Test (AVLT) immediate and delayed recall (Rey, 1958) as a measure of immediate and delayed memory. The AVLT immediate recall score is the sum of 5 learning trials of a 15 word list, scores range from 0 to 75. In the AVLT delayed recall, the 15 word list has to be recalled after 20 min, so scores range from 0 to 15. The Verbal Fluency Test (VFT) (Lezak et al., 2004) was used as a measure of verbal fluency, in which a person has to name as many animals as possible within 1 min. The Trail Making Test (TMT) part A was used as measure of attention, in which a person has to connect numbers (1–25) in ascending order, as quickly as possible (Reitan, 1979). TMT part B was used as a measure of executive functioning in which a person should alternate between connecting numbers and letters in ascending order, as quickly as possible. For TMT part A and B time in seconds was used as an outcome.

#### Biomarker analyses

CSF biomarker assessments of Aβ1-42, total tau (t-tau) and phosphorylated tau (p-tau) were performed according to the routine protocol in *n* = 569 individuals. For the four cohorts that used a PET scan to measure amyloid (*n* = 338), [^18^F]flutemetamol or [^18^F]AV45 PET scans were visually rated locally or the standardized uptake value (SUV) was calculated. We used center-specific cut-offs to define abnormal biomarker values (Supplemental Table 2). Amyloid positivity (Aβ+) was defined as an abnormal amyloid profile in CSF or on amyloid PET. Since the concordance between t-tau and p-tau was not similar across cohorts, tau positivity (T+) was defined as both abnormal t-tau and abnormal p-tau in CSF (Supplemental Table 2).

### Validation dataset

To test whether the new norms improved prediction of dementia in a clinical setting, we used an independent dataset from the Maastricht Alzheimer Centre Limburg (ACL) cohort (*n* = 1,070), including individuals from the ongoing, longitudinal study of patients referred to the Maastricht Memory Clinic (Visser et al., 2006). For the current study, we selected 1,070 individuals based on the following inclusion criteria were: (1) age above 40 years; (2) no dementia diagnosis at baseline; (3) baseline data on at least one the following measures: AVLT immediate recall, AVLT delayed recall, VFT, TMT-A, or TMT-B; and (4) minimally one clinical follow-up, at least 6 months after the baseline diagnosis.

In the validation dataset no biomarker information was used, only cognitive test performance and progression to dementia at follow-up. As the Maastricht ACL cohort is a memory clinic cohort it contains individuals with subjective or objective cognitive impairment.

#### Cognitive data

Cognitive data was available for the following neuropsychological tests: AVLT immediate and delayed recall, VFT, TMT-A, and TMT-B. Standardized scores (z-scores) based on published norms were already available as they are used in standard current clinical practice. These published norms were adjusted for age, gender, and years of education as described in previous publications: AVLT Immediate and Delayed recall (Van der Elst et al., 2005), VFT (Van der Elst et al., 2006), TMT part A and B (Schmand et al., 2003). In addition, we calculated novel z-scores based on biomarker negative norms derived from the test dataset.

#### Clinical diagnosis

For the current study we used a clinical diagnosis at follow-up that was made as part of standard clinical practice. Standard clinical practice includes: clinical interview, neuropsychological examination (raw scores and z-scores based on published norms) and magnetic resonance imaging (MRI). As CSF collection is not part of routine clinical practice this was usually not used when clinical diagnoses were made. Data on CSF biomarkers was available in a small subgroup (*n* = 104, 10%). Z-scores based on biomarker negative normative data were not available at the time of clinical diagnosis. Diagnosis of dementia at follow-up was made according to Diagnostic and Statistical Manual of Mental Disorders, Fourth Edition.(APA, 1994) Clinical etiological diagnoses for subtypes of dementia were made according to standardized clinical criteria for AD-type dementia (McKhann et al., 1984), vascular dementia (Roman et al., 1993), frontotemporal dementia (Neary et al., 1998), and Lewy body dementia (McKeith et al., 1996). As etiological diagnoses were made without CSF biomarkers, we used a generic dementia diagnosis as main outcome measure.

### Statistical analyses

#### Analyses in test dataset

As tau was only available in a subgroup of the test dataset, demographics were compared between Aβ- and Aβ+ groups, using *t-*tests for continuous and Chi-square for categorical variables. General linear mixed models, with random intercept at study level, were used to assess the influence of biomarkers on neuropsychological test performance using the following method: for each cognitive outcome measure (AVLT Immediate, AVLT Delayed, VFT, TMT-A, and TMT-B), we started with a standard model examining the influence of age, gender, and years of education on test performance (model 1). Next, we added Aβ status (model 2 = model 1 + Aβ) and tau status (model 3 = model 2 + tau).). In model 4, we entered all variables from model 3 and tested all two-way interactions between variables using a forward selection method. All non-significant interactions were removed from the model, significant interactions are shown in Table 2. Based on the significant predictors for each neuropsychological test, we calculated adjusted standardized scores (z-scores) using a regression-based approach based on only the biomarker negative individuals (Table 3). Age and year of education were entered as continues variables, sex as a dichotomous variable (female = 0, male = 1). For the TMT-A and TMT-B the regression formulas were multiplied by −1, such that a higher raw score indicated worse performance. We also performed sensitivity analyses regarding the method of defining Aβ status (PET vs. CSF) by adding a dichotomous variable (0 = CSF, 1 = PET) to the statistical models.

#### Analyses in validation dataset

**Figure 1:**
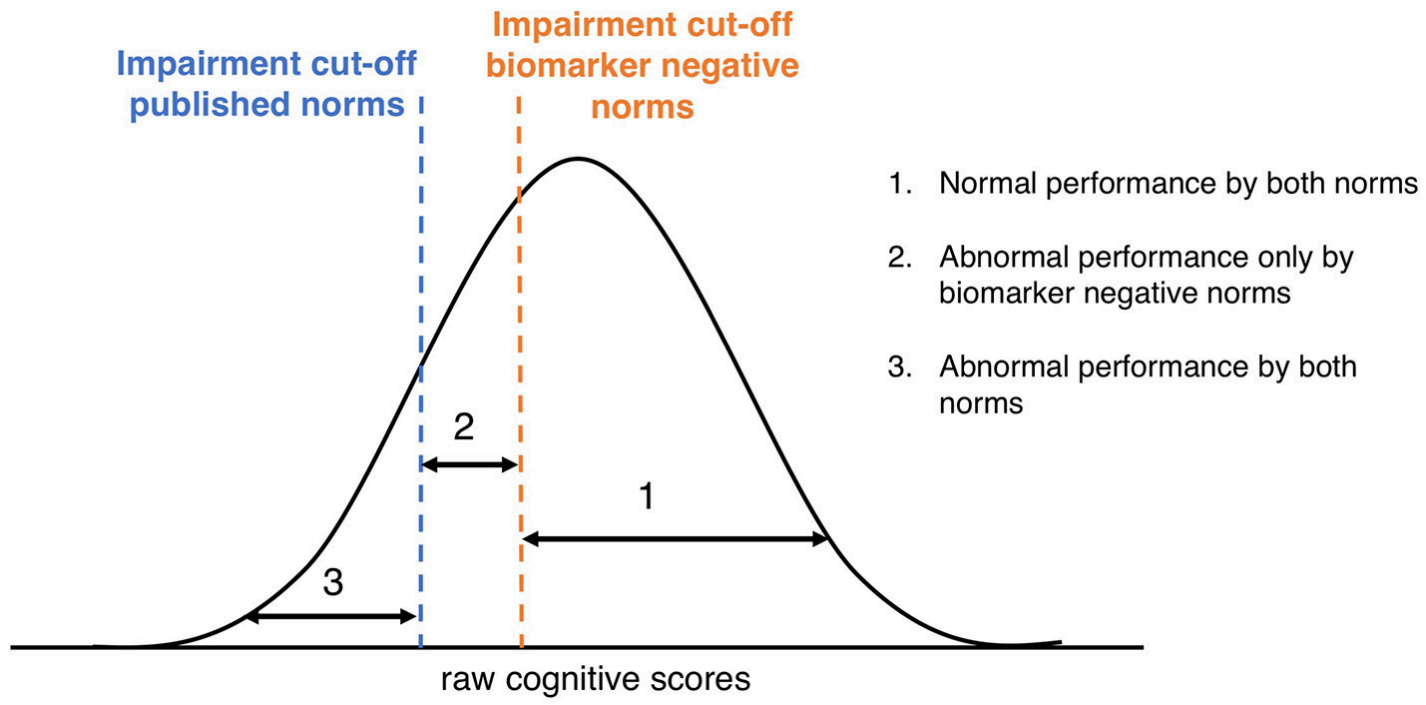
Classification according to published norms and Aβ- norms. Graphical representation of distribution of raw cognitive scores and the different cut-points using published norms or Aβ- norms.

We classified the subjects in the validation dataset into three groups as illustrated in Figure 1: (1) individuals with a z-score ≥-1.5 based on both published and Aβ- norms: “Normal performance by both norms”; (2) individuals with a z-score ≥-1.5 based on the published norms, but a z-score < -1.5 based on the Aβ- norms: “Abnormal performance only by biomarker negative norms”; (3) individuals with a z-score < -1.5 based on both published and Aβ- norms: “Abnormal by both norms.” Cox proportional hazard models were used to calculate the relative risk of progression to dementia for the three groups, adjusted for age, gender, and years of education. Since no biomarkers were required for the diagnosis of AD-type dementia in the validation cohort, we used a dementia diagnosis with unspecified etiology as the main outcome measure. Lastly, we used a 2 × 2 contingency table to calculate odds ratios, sensitivity, specificity, positive predictive value (PPV), and negative predictive value (NPV) of progression to dementia, given an abnormal performance according to the biomarker negative norms and the published norms. When determining the short-term predictive accuracy measures of progression to dementia, we used dementia within two years (dichotomous variable) as an outcome. Statistical analyses were conducted using R Statistical Software (version 3.3.3) and SPSS version 24.0 (Chicago, IL, USA) with significance set at *p* < 0.05.

## Results

### Test dataset

The test dataset consisted of 907 individuals with a mean age of 68.0 (*SD* 9.1) years and an average of 14.7 (*SD* 3.7) years of education. Four hundred and eighty-one (53%) of these were female. In 334 (37%) individuals, Aβ was measured using PET. In the remaining 568 (63%) individuals Aβ status was measured using CSF and these individuals also had data available on CSF tau status. Two hundred and twenty-nine (25%) were Aβ+. Of the *n* = 569 that had data on tau status available, *n* = 73 (8%) were T+.

Table 1 shows the demographic and cognitive variables for Aβ- and Aβ+ individuals. The Aβ+ group was significantly older (*p* < 0.001), more likely to carry an APOE ε4 allele (*p* < 0.001) and had more often abnormal t-tau and p-tau levels (*p* < 0.001), compared to the Aβ- group. The groups did not differ in gender distribution (*p* = 0.490), years of education (*p* = 0.907) or baseline MMSE score (*p* = 0.801). Regarding cognition, the Aβ- group outperformed the Aβ+ group on the AVLT Immediate (*p* = 0.020), AVLT delayed (*p* < 0.001), TMT-A (*p* = 0.011), and TMT-B (*p* < 0.001). There was no difference in performance on the VFT (*p* = 0.178; Table 1).

**Table 1:** Demographic and cognitive variables by Aβ status in test dataset.

	**No. Aβ-/Aβ+**	**Aβ-**	**Aβ+**	***p*-value**
**DEMOGRAPHICS**				
Age, years	677/230	66.7 (14.7)	71.8 (8.6)	< 0.001
Female, n	677/230	358 (53%)	128 (56%)	0.466
Education, years	677/230	14.7 (3.7)	14.7 (3.6)	0.907
APOE-ε4 carrier, n	629/218	174 (28%)	105 (48%)	< 0.001
MMSE, score	677/230	29.0 (1.1)	28.9 (1.1)	0.637
Abnormal CSF t-tau, n	398/175	42 (11%)	45 (26%)	< 0.001
Abnormal CSF p-tau, n	395/174	100 (25%)	85 (49%)	< 0.001
**COGNITIVE RAW SCORES**				
AVLT Immediate, words recalled	440/183	45.5 (10.1)	43.5 (9.4)	0.020
AVLT Delayed, words recalled	439/182	8.8 (3.4)	7.7 (3.6)	< 0.001
VFT, words named	526/190	21.6 (5.7)	20.9 (5.5)	0.178
TMT-A, seconds	674/230	36.7 (14.6)	39.8 (17.7)	0.011
TMT-B, seconds	670/228	85.5 (38.3)	99.6 (62.1)	< 0.001

*Results are mean (SD) or frequency (%): Aβ, amyloid-beta; APOE, Apolipoprotein E; AVLT, Auditory Verbal Learning Test; CSF, cerebrospinal fluid; MMSE, mini mental state examination; No, number; p-tau, phosphorylated tau; TMT, trail making test; t-tau, total tau; VFT, verbal fluency test*.

### Influence of Aβ and tau on cognition

Table 2 shows the outcomes of the linear mixed models which tested the influence of demographics and biomarkers on neuropsychological test performance. Age and education were associated with performance on all tests, and female gender was associated with a better performance on memory tests only (Table 2). Aβ status was a significant predictor for the TMT part A and part B (all *p* < 0.01). When tau was added to the models (model 3), the associations between tau status and test performance did not reach significance for any of the neuropsychological tests. In model 4, with 2-way interactions, we found that for the AVLT Immediate, AVLT Delayed and TMT-B the effect of Aβ depended on age, such that Aβ status predicted performance only at higher ages (model 4; interaction Aβ^*^age: AVLT Immediate *p* = 0.048; AVLT Delayed *p* = 0.025; TMT-B *p* = 0.030). The other 2-way interactions were all found insignificant. Additionally, we tested the influence of tau pathology on cognition without adding Aβ (model 3 without Aβ), and found no significant influence of tau on any of the cognitive tests (data not shown). The sensitivity analyses showed that the method of defining Aβ status (CSF vs. PET) did not have an influence on the results.

**Table 2:** Stepwise testing of linear mixed models including CSF biomarkers to predict cognitive test performance.

**Outcome**	**Model**	***n***	**Age**	**Gender**	**Education**	**Aβ**	**Tau**	Aβ^*^age
AVLT Immediate	Model 1	623	−0.44 ± 0.06^***^	−5.63 ± 0.73^***^	0.41 ± 0.11^***^			
	Model 2	623	−0.43 ± 0.06^***^	−5.64 ± 0.73^***^	0.40 ± 0.11^***^	−0.99 ± 0.82		
	Model 3	318	−0.38 ± 0.08^**^	−5.62 ± 1.09^***^	0.92 ± 0.19^***^	−0.26 ± 1.08	−0.74 ± 1.43	
	Model 4	318	−0.54 ± 0.11^***^	−5.67 ± 1.08^***^	0.91 ± 0.19^***^	20.51 ± 10.53^*^	−0.33 ± 1.44	−0.29 ± 0.14^*^
AVLT delayed	Model 1	621	−0.12 ± 0.02^***^	−1.49 ± 0.26^***^	0.07 ± 0.04			
	Model 2	621	−0.12 ± 0.02^***^	−1.49 ± 0.26^***^	0.07 ± 0.04	−0.41 ± 0.30		
	Model 3	317	−0.14 ± 0.03^**^	−1.09 ± 0.42^*^	0.17 ± 0.08^*^	−0.27 ± 0.41	−0.61 ± 0.55	
	Model 4	317	−0.20 ± 0.04^***^	−1.10 ± 0.41^**^	0.16 ± 0.07^*^	8.87 ± 4.08^*^	−0.43 ± 0.55	−0.13 ± 0.06^*^
VFT	Model 1	716	−0.16 ± 0.03^***^	−0.20 ± 0.41	0.36 ± 0.06^***^			
	Model 2	716	−0.16 ± 0.03^***^	−0.18 ± 0.41	0.35 ± 0.06^***^	0.41 ± 0.48		
	Model 3	414	−0.20 ± 0.04^***^	−0.07 ± 0.53	0.39 ± 0.08^***^	0.60 ± 0.56	−0.50 ± 0.75	
TMT-A	Model 1	904	0.69 ± 0.06^***^	0.81 ± 0.85	−0.71 ± 0.12^***^			
	Model 2	904	0.67 ± 0.06^***^	0.89 ± 0.85	−0.70 ± 0.12^***^	2.25 ± 1.01^**^		
	Model 3	567	0.61 ± 0.08^***^	−0.50 ± 1.08	−0.75 ± 0.17^***^	3.53 ± 1.21^**^	−0.73 ± 1.63	
TMT-B	Model 1	898	1.70 ± 0.16^***^	2.27 ± 2.22	−2.20 ± 0.32^***^			
	Model 2	898	1.63 ± 0.16^***^	2.52 ± 2.21	−2.17 ± 0.32^***^	7.47 ± 2.61^**^		
	Model 3	563	1.48 ± 0.20^***^	−0.66 ± 2.77	−2.16 ± 0.43^***^	10.24 ± 3.11^**^	−2.38 ± 3.11	
	Model 4	563	1.97 ± 0.30^***^	−0.71 ± 2.77	−2.16 ± 0.43^***^	−38.73 ± 22.74	−3.39 ± 4.17	0.70 ± 0.32^*^

*Numbers are mixed model coefficients ± standard error*.

* *p < 0.05*,

** *p < 0.01*,

*** *p < 0.001. Model 4 was only added when any of the two-way interactions were significant. Aβ, amyloid-beta; AVLT, Auditory Verbal Learning Test; TMT-A, Trail Making Test part A; TMT-B, Trail Making Test part B; VFT, verbal fluency test*.

### Aβ- norms

Next, we calculated standardized normative scores (z-scores) for the AVLT Immediate and Delayed recall and the TMT part A and B based on Aβ- individuals only. Table 3 shows the equations to calculate the z-scores for the four cognitive measures, based on regression analyses.

**Table 3:** Z-scores equations based on Aβ- individuals.

**Neuropsychological test**	**Raw score range**	***n***	**Z-score equations**
AVLT Immediate	0–75	440	Z-score = Raw score–(68.396 + −0.363 * AGE + −5.913 * SEX + 0.358 * EDUCATION)/3.048
AVLT delayed	0–15	439	Z-score = Raw score–(17.508 + −0.117 * AGE + −1.586 * SEX + 0.019 * EDUCATION)/1.796
TMT-A	15–100	674	Z-score = −1*(Raw score–(20.020 + 0.433 * AGE + −0.839 * EDUCATION)/3.587)
TMT-B	20–200	670	Z-score = −1*(Raw score–(37.609 + 1.251 * AGE + −2.486 * EDUCATION)/5.627)

*AVLT Immediate, Auditory Verbal Learning Test sum of 5 learning trials; AVLT Delayed, Auditory Verbal Learning Test delayed recall; TMT-A, Trail Making Test part A; TMT-B, Trail Making Test part B. Variables are coded as follows: Age (continuous variable); Sex: Female = 0, Male = 1; Education: years of education (continuous variable)*.

### Validation dataset

To test the prognostic utility of the norms based on Aβ- individuals, we used a validation cohort of 1,070 memory clinic visitors with subjective or objective cognitive deficits. Table 4 shows the characteristics of the validation cohort. The average age was 63.2 (*SD* 10.7) years and 41% were female (Table 4). Five hundred forty-seven (51%) were cognitively impaired in at least one cognitive domain, according to published norms. The average follow-up was 5.2 (*SD* 3.8) years and at the last follow-up visit, 255 (24%) had progressed to dementia (Table 4).

**Table 4:** Characteristics of validation dataset.

**Characteristics**	**Total sample**
n	1,070
Age, years	63.2 (10.7)
Female, n	437 (41%)
Education, years	10.5 (3.1)
Follow-up length, years	5.2 (3.8)
Progression to dementia, n	251 (24%)
•[-] AD-type dementia, n	170 (16%)
•[-] Vascular dementia, n	21 (2%)
•[-] Frontotemporal dementia, n	5 (1%)
•[-] Lewy Body or Parkinson dementia, n	5 (1%)
•[-] Other dementia or unknown etiology, n	50 (5%)
Impairment in only one cognitive domain*, n	328 (31%)
Impairment in multiple cognitive domains*, n	219 (20%)
MMSE, score	27.9 (2.2)
AVLT Immediate, words recalled	37.9 (11.4)
AVLT Delayed, words recalled	6.9 (3.8)
VFT, words named	19.9 (6.3)
TMT-A, seconds	50.0 (19.0)
TMT-B, seconds	91.1 (44.8)

Results are mean (SD) or frequency (%). ^*^Based on z-score < -1.5 derived from published normative data. AD, Alzheimer's disease; AVLT, Auditory Verbal Learning Test; MMSE, Mini Mental State Examination; TMT, Trail Making Test; VFT, Verbal Fluency Test.

### Prognostic utility of Aβ- norms

**Figure 2:**
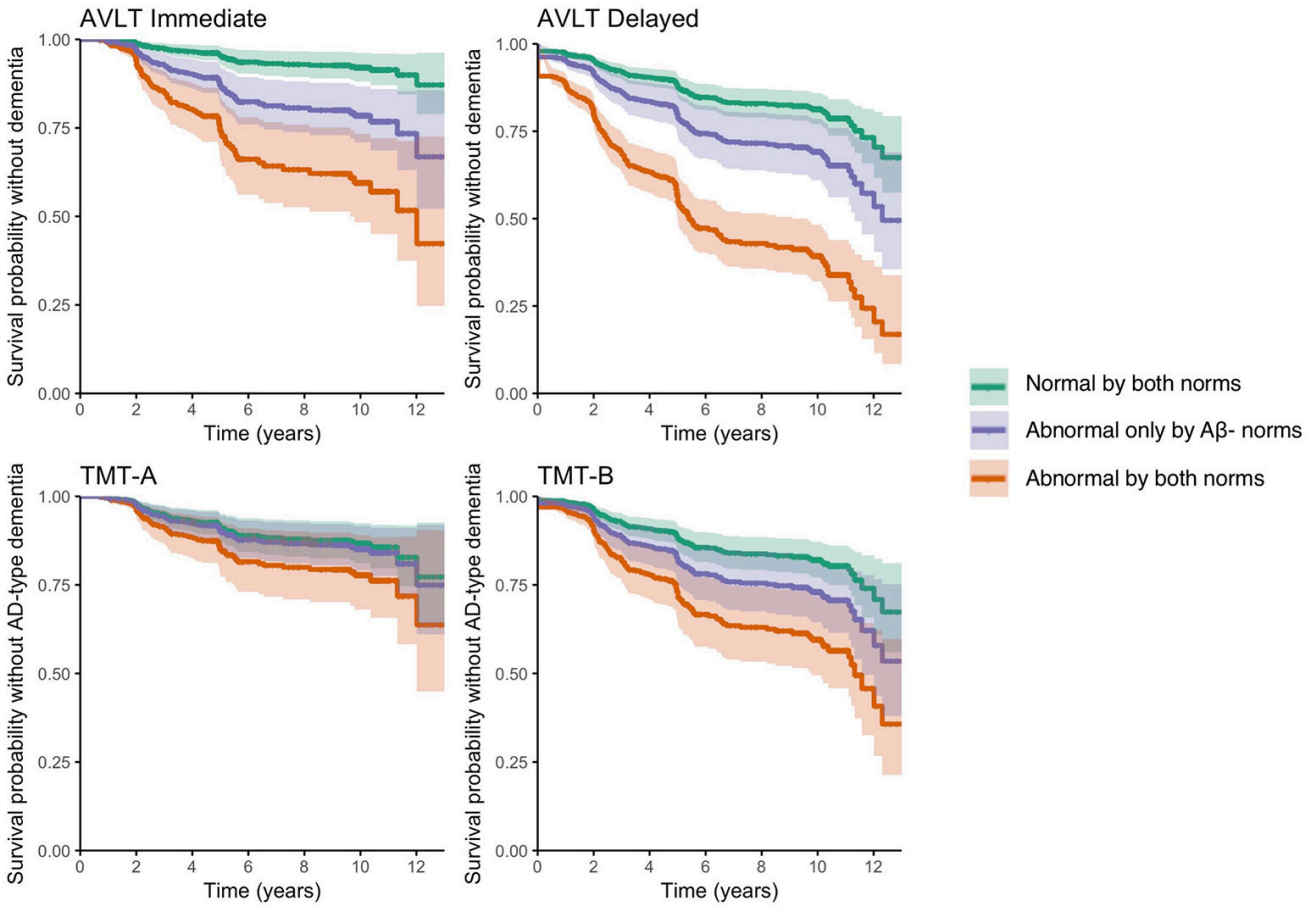
Progression to dementia for groups classified by current and Aβ- normative data in validation dataset. Survival probability without dementia for individuals who perform normal according to both norms (group 1), individuals who perform abnormal only according to Aβ- norms (group 2), individuals who perform abnormal according to both norms (group 3) on the AVLT Immediate **(top left)**, AVLT Delayed **(top right)**, TMT-A **(bottom left)**, and TMT-B **(bottom right)**. Survival curves are adjusted for age, gender, and education.

Table 5 shows the classifications (normal/abnormal) according to the Aβ- norms and the published norms for the AVLT Immediate, AVLT Delayed, TMT-A, and TMT-B. Depending on the assessed test, 38–64% of the individuals had a normal performance according to both norms, 16–38% had an abnormal performance only according to the Aβ- norms and 17–31% had an abnormal performance according to both norms. Individuals who performed abnormal according to both norms on the AVLT Immediate, AVLT Delayed, TMT-A, and TMT-B showed a faster progression rate to dementia, compared to individuals who performed normal according to the Aβ- norms (*p* < 0.001), and those performing abnormal according to both norms (*p* < 0.001; Table 5; Figure 2). In addition, individuals who performed abnormal only according to the Aβ- norms on the AVLT Immediate and AVLT delayed, progressed at a faster rate to dementia relative to individuals who performed normal according to both norms (AVLT Immediate *p* < 0.001; AVLT Delayed *p* = 0.009; Table 5; Figure 2). We also calculated hazard ratios for an abnormal performance, relative to a normal performance, for published norms and Aβ- norms (Supplemental Table 3).

**Table 5:** Progression rate to dementia during follow-up for groups classified by published and Aβ- norms in validation dataset.

**Test**	**(1) Normal performance by both norms**		**(2) Abnormal performance only by A**β**- norms**				**(3) Abnormal performance by both norms**			
	***n***	**HR**	***n***	**HR**	**95% CI**	***p*-values group comparisons**	***n***	**HR**	**95% CI**	***p*-values group comparisons**
AVLT Immediate	403 (38%)	Ref	387 (36%)	2.58	1.8–3.7	(1) < 0.001; (3) < 0.001	280 (26%)	4.76	3.3–6.9	(1) < 0.001
AVLT Delayed	568 (53%)	Ref	167 (16%)	1.77	1.2–2.7	(1) 0.009; (3) < 0.001	335 (31%)	4.30	3.2–5.8	(1) < 0.001
TMT-A	304 (42%)	Ref	278 (38%)	1.20	0.8–1.7	(1) 0.298; (3) 0.001	145 (20%)	2.25	1.5–3.4	(1) < 0.001
TMT-B	419 (64%)	Ref	121 (19%)	1.30	0.9–1.9	(1) 0.197; (3) 0.003	114 (17%)	2.63	1.8–3.9	(1) < 0.001

*Hazard Ratios (HR) and 95% Confidence intervals (CI) are calculated with the unimpaired group as a reference, adjusted for age, gender, and years of education: Aβ, amyloid- beta; AVLT Immediate, Auditory Verbal Learning Test sum of 5 learning trials; AVLT Delayed, Auditory Verbal Learning Test delayed recall; TMT-A, Trail Making Test part A; TMT-B, Trail Making Test part B; Ref, reference group. (1) p-value compared to group 1; (3) p-value compared to group 3*.

### Short term predictive accuracy of Aβ- and published norms

During two years of follow-up, 144 (14%) of the individuals in the validation dataset progressed to dementia. Table 6 shows the short-term predictive accuracy measures of progression to dementia within two years since baseline visit, given an abnormal performance using the Aβ- norms or the published norms. When predicting risk of progression to dementia, odds were higher for Aβ- norms for AVLT Immediate, TMT-A and TMT-B compared to published norms. For the AVLT Delayed, the odds of progression to dementia were higher for published norms compared to Aβ- norms. The Aβ- norms had a higher sensitivity, but a lower specificity compared to the published norms for all cognitive tests (Table 6). We also calculated the predicative accuracy of being Aβ+ in the test dataset, results were comparable to the results with progression to dementia as an outcome (Supplemental Table 4).

**Table 6:** Predictive accuracy of progression to dementia within 2 years given an abnormal performance using Aβ- or published norms.

**Test**	**Norms**	***n* Normal/abnormal**	**OR**	**Sensitivity**	**Specificity**	**PPV**	**NPV**
AVLT Immediate	Aβ- norms	406/664	4.72 (2.9–7.8)	0.87 (0.8–0.9)	0.42 (0.4–0.5)	0.19 (0.2–0.22)	0.95 (0.9–0.97)
	Published norms	790/280	3.45 (2.4–5.0)	0.50 (0.4–0.6)	0.78 (0.7–0.8)	0.26 (0.2–0.3)	0.91 (0.9–0.93)
AVLT Delayed	Aβ- norms	570/500	4.26 (2.8–6.4)	0.76 (0.7–0.8)	0.58 (0.5–0.6)	0.22 (0.2–0.3)	0.94 (0.9–0.96)
	Published norms	735/335	6.43 (4.4–9.4)	0.69 (0.6–0.8)	0.75 (0.7–0.8)	0.30 (0.2–0.3)	0.94 (0.9–0.95)
TMT-A	Aβ- norms	306/427	1.78 (1.1–2.9)	0.70 (0.6–0.8)	0.43 (0.4–0.5)	0.15 (0.1–0.2)	0.91 (0.9–0.94)
	Published norms	582/145	1.38 (0.8–2.3)	0.25 (0.2–0.3)	0.81 (0.8–0.84)	0.16 (0.1–0.2)	0.88 (0.85–0.9)
TMT-B	Aβ- norms	423/237	2.83 (1.8–4.5)	0.58 (0.5–0.7)	0.67 (0.6–0.7)	0.20 (0.1–0.3)	0.92 (0.9–0.95)
	Published norms	539/155	1.84 (1.1–3.0)	0.33 (0.2–0.4)	0.79 (0.76–0.8)	0.19 (0.1–0.2)	0.89 (0.86–0.9)

*Numbers are estimates with (95% CI): Aβ, amyloid-beta; AVLT, Auditory Verbal Learning Test; NPV, negative predictive value; OR, odds ratio; PPV, positive predictive value; TMT-A, Trail Making Test part A; TMT-B, Trail Making Test part B*.

### *Post-hoc* analyses

When repeating the survival analyses with progression to clinical diagnosis of AD-type dementia as an outcome, associations were similar (data not shown). Results were also comparable after exclusion of individuals for whom CSF biomarker data was available when the clinical diagnosis was made (*n* = 104). As we found in the analyses in the test cohort that age was related to the influence of Aβ on cognition, we also repeated the validation analyses in younger (≤ 70) and older (>70) individuals separately. Associations also remained similar when stratifying by age (data not shown).

## Discussion

In a large cohort of cognitively normal individuals we tested the influence of Aβ and tau biomarkers on cognitive test performance. We found that Aβ influenced performance on all assessed cognitive tests, except for the VFT. The additional presence of tau did not further influence performance in any cognitive domain. For memory tests, we found that normative data based on only Aβ- individuals was more sensitive in identifying individuals at risk of a faster progression to dementia. For non-memory tests, we found no difference between the Aβ- norms and published norms.

### Aβ, tau, and cognition

Our study provides additional evidence that in cognitively normal individuals an abnormal Aβ biomarker negatively influences cognitive performance in the domains of memory, attention and executive functioning (Mielke et al., 2016; Petersen et al., 2016; Baker et al., 2017). However, effects were small to moderate in particular in individuals below 70 years of age, which could explain why previous studies with smaller sample sizes and younger individuals reported no associations between Aβ and cognition (Hedden et al., 2013). We found that tau did not influence cognitive test performance in addition to Aβ, nor in absence of Aβ. Moreover, we found that the influence of Aβ on cognition was not influenced by tau status (i.e., Aβ-tau interaction). These findings are incongruent with previous findings in individuals with MCI as well as in cognitively normal individuals showing that tau and other neurodegenerative features, rather than Aβ, are associated with cognitive impairment and decline in various cognitive domains (Nelson et al., 2012; Mormino et al., 2014; Pettigrew et al., 2015; Brier et al., 2016; Degerman Gunnarsson et al., 2016; Dumurgier et al., 2017; Cerami et al., 2018). However, there are noteworthy differences between these previously mentioned studies and our study in determining tau status (CSF vs. PET), cognitive outcome measure used (single test, composite score, or computerized test) and design of the study (cross-sectional cognitive performance vs. longitudinal cognitive decline). Taken together, this may imply that the influence of abnormal CSF tau on baseline cognition performance on the tests that we investigated may be detectable only from the prodromal AD stage onwards (i.e., when cognitive impairments are already present). Future studies in different stages of the disease are needed to validate this notion.

### Prognostic utility of Aβ- norms

When comparing three groups classified by Aβ- norms and published norms in their progression rate to dementia, we showed that excluding Aβ+ individuals from normative data for memory tests increased the predictive accuracy for future progression to dementia. This seems to conflict with findings from a previous study (Hassenstab et al., 2016). However, in this previous study they investigated combined Aβ- and tau- norms, instead of only Aβ- norms, and validated the norms in a preclinical cohort with progression to CDR ≥ 0.5 as an outcome, whereas we validated our norms in a large memory clinic cohort with progression to dementia as an outcome. For non-amnestic cognitive tests (i.e., TMT-A and TMT-B), we found that Aβ pathology was associated with a worse performance, but the prognostic utility of the Aβ- norms was comparable to published norms. This implies that although Aβ pathology influences performance in non-amnestic domains in the preclinical stage, only a severe non-amnestic cognitive impairment (i.e., impairment according to published norms) is predictive of progression to dementia.

### Sensitivity vs. specificity

While we showed that individuals who performed normal by published but abnormal by Aβ-norms on memory tests (group 2) progressed faster to dementia than individuals who performed normal by Aβ- norms (group 1) during 12 years of follow-up, application of the Aβ- norms only slightly improved short-term predictive accuracy (2 years) compared to published norms (AVLT immediate, TMT A & B). This could indicate that two years is too short as a follow-up or that the increase in sensitivity in detecting those at risk of progression to dementia by the Aβ- norms, is accompanied by a decrease in specificity (i.e., individuals with abnormal scores according to Aβ- norms who will not progress to dementia). It is however important to note that we only compared different normative data to each other in their predictive accuracy of dementia and did not compare the predictive value of the cognitive tests. Hence, it cannot be deduced from our results which neuropsychological test is the best predictor of AD. Based on the comparisons between the published and Aβ- norms, we recommend to use the Aβ- norms in combination with published norms by creating three groups of memory classification instead of two, as illustrated in Figure 1, since this will improve prognostic accuracy for individual patients. For example, a 70-year old male with 16 years of education who recalled between 29 and 38 words on the AVLT Immediate would be classified in group 2 (normal by published, abnormal by Aβ- norms), implying that he is at intermediate risk of progression to dementia. Should he have recalled < 29 words he would have been classified in group 3 (abnormal by published norms) with the highest risk of progression, and in case he recalled more than 38 words he would have been classified in group 1 (normal by Aβ- norms) with the lowest risk of progression.

### Age

Age was a consistent predictor of cognitive performance in all cognitive domains even after accounting for present AD pathology, confirming earlier findings (Oh et al., 2012; Hassenstab et al., 2016). Interestingly, older age augmented the effect of Aβ on memory and executive functioning, which is in line with previous studies (Lim et al., 2016; Jansen et al., 2017). It is possible that at higher ages amyloid pathology is more extensive and thereby has a larger effect on cognition. Moreover, in older individuals amyloid pathology more often co-exists with other pathologies and together these pathologies may have an additive or synergistic effect on cognition, possibly through processes like inflammation (Franceschi and Campisi, 2014; Vemuri and Knopman, 2016). Inflammation has been associated with many concomitant pathologies, but also with Aβ itself, and has shown to affects cognition, in particular at higher age (Giunta et al., 2008; Simen et al., 2011).

### Strengths and limitations

Strengths of the current study are the large sample sizes of both the test and validation datasets, assessment of commonly used neuropsychological tests and a long follow-up time in the validation cohort. Nevertheless, the results of the current study must be understood in the context of several limitations. In the test dataset, we included subjects from different centers, which might have led to variability in the data. In addition, it is likely that there was some form of selection bias in the test dataset, making it less comparable to other normative dataset which are usually a reflection of the general population. However, the test data set did not contain individuals with cognitive complaints or individuals from clinical settings. We used only five neuropsychological tests as outcome measure, using additional test might have yielded different results. Our test sample was relatively young, which makes the Aβ- norms less reliable to assess cognition at higher ages. We used different methods for defining Aβ pathology (CSF and PET) and we only had data on tau available in a subgroup (e.g., the group with CSF). However, sensitivity analyses showed no influence of the method assessing Aβ and on the associations between Aβ+ and cognition and these remained similar when testing them in subgroups with CSF (tau) data or PET data available (data not shown). Although the biomarker collection (CSF and PET) was performed according to local routine collection protocols, local cut-off points were used to define Aβ status which is a frequently used method to pool biomarker data across studies (Jansen et al., 2015; Vos et al., 2015; Bos et al., 2017). Lastly, we only studied tau in CSF as a marker for neurodegeneration. Future studies should determine whether other neurodegenerative markers, like hippocampal atrophy on MRI or tau PET, might provide different results.

## Conclusion

Using the Aβ- norms has implications when assessing memory in clinical practice as well as in research settings. A disadvantage of using norms that do not take amyloid status into account (i.e., published norms) is that there is a substantial part of individuals that would be classified as having normal memory, while they may have underlying AD. Consequently it seems best to classify memory performance into three categories by combining Aβ- norms with published norms (Figure 1), rather than just classifying individuals as normal or abnormal by a single set of norms. The use of three groups for memory performance classification will improve diagnostic and prognostic accuracy for individual patients. Future studies should determine the diagnostic and prognostic values of using Aβ- norms for other cognitive tests.

## Ethics statement

Each study was approved by the local ethics committee and all subjects gave written informed consent in accordance with the Declaration of Helsinki.

## Author contributions

IB, SV, and PV: study concept and design; All authors: acquisition and/or interpretation of data and critical revision of final draft of manuscript; IB, SV, WJ, and PV: statistical analysis and drafting the manuscript.

### Conflict of interest statement

The authors declare that the research was conducted in the absence of any commercial or financial relationships that could be construed as a potential conflict of interest.
