# Supplementary material for: Amyloid-β, Tau, and Cognition in Cognitively Normal Older Individuals: Examining the Necessity to Adjust for Biomarker Status in Normative Data
Source: Front Aging Neurosci. 2018 Jun 25;10:193. doi: 10.3389/fnagi.2018.00193 (PMC6027060; doi:10.3389/fnagi.2018.00193)
Supplement: Supplementary file 2 [file Table_2.docx]

| **Supplemental table 2. Number of subjects in test dataset per center and biomarker protocol information** | | | | |  |  |
| --- | --- | --- | --- | --- | --- | --- |
| Cohorts | Definition of CN | n | Method of amyloid measurement | Biomarker cut-offs | | |
|  |  |  |  | Aβ | P-tau | T-tau |
| ADNI | CDR=0, no memory complaints, above cut-off on LM and MMSE ≥ 24 | 298 | CSF – xMAP Luminex / Amyloid-PET- [^18^F]AV45 | Aβ_1-42_ <192  SUV >1.1186 | >23 | >93 |
| Barcelona St Pau | GDS=1 and ->1.5 SD on FCSRT | 70 | CSF – Innotest Fujirebio-Europe | Aβ_1-42_ <550 | >61 | >350 |
| EDAR | On all assessed tests >-1.5 SD and no subjective memory complaints. | 34 | CSF – xMAP Luminex | Aβ_1-42_ <389 | >35 | >98 |
| EMIF preAD | CDR=0 and memory score >-1.5 SD | 100 | Amyloid-PET- [^18^F]flutemetamol | Visual rating |  |  |
| GAP | Scaled scores <7, CDR=0 | 112 | CSF – Innotest Fujirebio-Europe | Aβ_1-42_ <550 | >61 | >350 |
| Gothenburg | GDS=1 and >-1.5 SD on all assessed tests. | 47 | CSF – Innotest Fujirebio-Europe | Aβ_1-42_ <550 | Age <60: >60  Age ≥ 60: 80 | >400 |
| IDIBAPS | CDR=0 and ->1.5 SD on FCSRT | 35 | CSF – Innotest Fujirebio-Europe | Aβ_1-42_ <550/<500* | >75 | >350 |
| IMAP+ | >-1.65 SD on all assessed tests. Living at home with no memory complaints | 32 | Amyloid-PET - [^18^F]AV45 | SUV >1.0054 |  |  |
| Leuven | CDR=0, >-1.9 SD on all assessed tests, MMSE ≥ 27 | 179 | Amyloid-PET- [^18^F]flutemetamol | SUV >1.38 |  |  |
| *Cut-off value is different since samples were analyzed in two batches. Abbreviations: Aβ = amyloid-beta, CDR = Clinical Dementia Rating, CSF = cerebrospinal fluid, FCSRT = Free and Cued Selective Reminding Test, LM = Logical Memory Test, P-tau = phosphorylated tau, SUV = standardized uptake value, t-tau = total tau. | | | | | | |
