# Supplementary material for: Amyloid-β, Tau, and Cognition in Cognitively Normal Older Individuals: Examining the Necessity to Adjust for Biomarker Status in Normative Data
Source: Front Aging Neurosci. 2018 Jun 25;10:193. doi: 10.3389/fnagi.2018.00193 (PMC6027060; doi:10.3389/fnagi.2018.00193)
Supplement: Supplementary file 3 [file Table_3.DOCX]

| **Supplemental Table 2. Risk of progression to dementia for abnormal performance based on published and Aβ- norms** | | | | | | | |
| --- | --- | --- | --- | --- | --- | --- | --- |
|  | **Abnormal performance by**  **Published norms** | | |  | **Abnormal performance by**  **Aβ- norms** | | |
| **Test** | HR | 95% CI | p-value |  | HR | 95% CI | p-value |
| AVLT Immediate | 2.66 | 2.1 – 3.4 | <0.001 |  | 3.38 | 2.4 – 4.7 | <0.001 |
| AVLT Delayed | 3.73 | 2.9 – 4.8 | <0.001 |  | 3.43 | 2.6 – 4.6 | <0.001 |
| TMT-A | 2.03 | 1.4 – 2.9 | <0.001 |  | 1.43 | 1.0 – 2.0 | 0.028 |
| TMT-B | 1.68 | 1.2 – 2.3 | 0.002 |  | 1.65 | 1.2 – 2.3 | 0.003 |
| HR are calculated with normal performing group, according to used norms, as a reference. Abbreviations: Aβ = amyloid-beta, AVLT = Auditory Verbal Learning Test, CI = confidence interval, HR = hazard ratio, TMT = Trail Making Test. | | | | | | | |
