# Supplementary material for: Amyloid-β, Tau, and Cognition in Cognitively Normal Older Individuals: Examining the Necessity to Adjust for Biomarker Status in Normative Data
Source: Front Aging Neurosci. 2018 Jun 25;10:193. doi: 10.3389/fnagi.2018.00193 (PMC6027060; doi:10.3389/fnagi.2018.00193)
Supplement: Supplementary file 4 [file Table_4.DOCX]

| **Supplemental Table 3. Predictive accuracy of being** **Aβ+ given an abnormal performance using Aβ- or published norms** | | | | | | |
| --- | --- | --- | --- | --- | --- | --- |
| Test | Norms | OR | Sens | Spec | PPV | NPV |
| AVLT Immediate | Aβ- norms | 1.30 (0.9 – 1.9) | 0.38 (0.3 – 0.5) | 0.68 (0.6 – 0.7) | 0.33 (0.3 – 0.4) | 0.73 (0.7 – 0.8) |
|  | Pub. norms | 0.9 (0.4 – 2.3) | 0.04 (.01 - .07) | 0.96 (0.9 – 0.98) | 0.28 (0.1 – 0.5) | 0.71 (0.7 – 0.74) |
| AVLT Delayed | Aβ- norms | 1.45 (0.96 – 2.2) | 0.81 (0.8 – 0.84) | 0.26 (0.2 – 0.3) | 0.36 (0.3 – 0.4) | 0.72 (0.7 – 0.8) |
|  | Pub. norms | 1.96 (1.2 – 3.1) | 0.21 (0.1 – 0.3) | 0.88 (0.85 – 0.9) | 0.42 (0.3 – 0.5) | 0.73 (0.7 – 0.8) |
| TMT-A | Aβ- norms | 1.07 (0.8 – 1.5) | 0.28 (0.2 – 0.3) | 0.73 (0.7 – 0.8) | 0.26 (0.2 – 0.3) | 0.75 (0.7 – 0.8) |
|  | Pub. norms | 0.98 (0.5 – 2.1) | 0.04 (.01 – 0.1) | 0.96 (0.9 – 0.97) | 0.25 (0.1 – 0.4) | 0.75 (0.7 – 0.8) |
| TMT-B | Aβ- norms | 1.05 (0.8 – 1.4) | 0.32 (0.3 – 0.4) | 0.69 (0.6 – 0.7) | 0.26 (0.2 – 0.3) | 0.75 (0.7 – 0.8) |
|  | Pub. norms | 0.99 (0.6 – 1.6) | 0.10 (0.1 – 0.14) | 0.90 (0.9 – 0.92) | 0.25 (0.2 – 0.3) | 0.75 (0.7 – 0.8) |
| Numbers are estimates with (95% CI). Abbreviations: Aβ = amyloid-beta, AVLT = Auditory Verbal Learning Test, NPV = negative predictive value, OR = odds ratio, PPV = positive predictive value, Pub. = Published, TMT-A = Trail Making Test part A, TMT-B = Trail Making Test part B | | | | | | |
